# Amantadine Attenuates Secondary Oxidative and Inflammatory Injury by Modulating the HIF-1α/BNIP3L/HMGB1 Axis in Rat Model of Traumatic Brain Injury

**DOI:** 10.3390/medicina62020362

**Published:** 2026-02-11

**Authors:** Ahmet Bindal, Pinar Karabacak, Halil Asci, Ilter Ilhan, Muhammet Yusuf Tepebasi, Orhan Imeci, Ahmet Yunus Hatip, Ozlem Ozmen

**Affiliations:** 1 Department of Anesthesiology and Reanimation, Faculty of Medicine, Suleyman Demirel University, Isparta 32260, Turkey; pinarkarabacak@sdu.edu.tr; 2 Department of Pharmacology, Faculty of Medicine, Suleyman Demirel University, Isparta 32260, Turkey; halilasci@sdu.edu.tr (H.A.); orhanimeci@sdu.edu.tr (O.I.); 3 Department of Biochemistry, Faculty of Medicine, Suleyman Demirel University, Isparta 32260, Turkey; ilterilhan@sdu.edu.tr; 4 Department of Genetics, Faculty of Medicine, Suleyman Demirel University, Isparta 32260, Turkey; muhammettepebasi@sdu.edu.tr; 5 Department of Emergency Medicine, Faculty of Medicine, Suleyman Demirel University, Isparta 32260, Turkey; ahmethatip@sdu.edu.tr; 6 Department of Pathology, Faculty of Veterinary Medicine, Burdur Mehmet Akif Ersoy University, Burdur 15030, Turkey; ozlemo@mehmetakif.edu.tr

**Keywords:** central nervous system, N-methylaspartate antagonists, mitophagy, mitochondrial disorders, oxidative stress

## Abstract

*Background and Objectives:* Traumatic brain injury (TBI) triggers oxidative stress, mitochondrial dysfunction, and sterile inflammation. Amantadine (ATD), a weak NMDA receptor antagonist, has shown neuroprotective potential, but its mechanistic basis remains unclear. This study examined whether ATD treatment is associated with changes in molecular and histological markers related to the *HIF-1α*/*BNIP3L*/*HMGB1*-mediated hypoxia–mitophagy–inflammation response in a rat TBI model. *Materials and Methods:* Thirty-two Wistar rats were assigned to four groups: sham, trauma, trauma + ATD (1 day), and trauma + ATD (7 days). TBI was induced using the impact-acceleration model, and ATD (45 mg/kg, i.p.) was administered post-injury. Oxidative stress indices (TOS, TAS, OSI), histopathology, inflammatory/apoptotic markers (CRP, TNF-α, Caspase-3), and gene expression (*HIF-1α*, *BNIP3L*, *HMGB1*) were evaluated. *Results:* ATD improved oxidative balance and histopathological integrity while reducing TNF-α, CRP, and Caspase-3 immunoreactivity. qPCR analysis showed lower *HIF-1α*, *BNIP3L*, and *HMGB1* expression in ATD-treated groups, which is consistent with attenuation of hypoxia-related, mitochondrial stress-associated, and damage-associated molecular pattern-associated signaling after injury. *Conclusions:* In this experimental model, amantadine ameliorated oxidative, inflammatory, and apoptotic markers and was associated with reduced expression of HIF-1α, BNIP3L, and HMGB1. These findings support a mechanistic correlation between ATD treatment and suppression of secondary injury signatures; however, causal pathway relationships and functional neurological outcomes were not assessed.

## 1. Introduction

Traumatic brain injury (TBI) is one of the leading causes of death and permanent neurological impairment globally. TBI, which is one of the leading causes of morbidity, especially in young adults, accounts for approximately one-quarter of all trauma-related deaths [1,2]. The severity of the trauma, the presence of systemic inflammation, and accompanying organ injuries significantly increase mortality and lead to long-term cognitive and neurological sequelae in survivors [3].

The pathophysiology of TBI is divided into two main phases. Primary injury results from mechanical forces, while secondary injury involves molecular processes that begin within minutes and can persist for days. The main molecular processes include excitotoxicity, oxidative stress, mitochondrial dysfunction, neuroinflammation, and apoptosis [4,5]. One of the key triggers of secondary damage is glutamate-mediated N-methyl-D-aspartate (NMDA) receptor activation. Post-traumatic cellular energy loss, mitochondrial dysfunction, and membrane depolarization lead to excessive glutamate release from presynaptic terminals. This situation leads to calcium influx into the cell via NMDA receptors, disruption of ionic balance, and irreversible neuronal damage [6]. Disruption of the blood–brain barrier (BBB) and loss of ionic homeostasis also exacerbate this process [7].

Amantadine (ATD) is a prominent agent in this context, acting as a weak NMDA receptor antagonist and an indirect dopamine agonist. It is used in clinical practice to support alertness and cognitive recovery in cases of impaired consciousness [8]. However, recent studies have shown that the neuroprotective effects of ATD after trauma are not limited to NMDA blockade; it may also be effective on mitochondrial quality control, the hypoxic response, and inflammatory signaling pathways [7,9,10]. Clinical and translational evidence, including recent mechanistic reviews, supports the broader neuroprotective potential of ATD after brain injury beyond NMDA antagonism, with reported anti-inflammatory and mitochondrial-associated effects [8,9].

Post-traumatic oxidative stress is also one of the key components of secondary brain injury. Increased reactive oxygen species (ROS) and decreased antioxidant defense disrupt cellular integrity. Laboratory indicators of this balance include Total Oxidant Status (TOS), Total Antioxidant Status (TAS), and their ratio, the Oxidative Stress Index (OSI). Elevated TOS and OSI values and decreased TAS levels in TBI models have revealed the significant role of oxidative stress in neurodegeneration [11,12].

Preserving mitochondrial integrity is critical for neuronal survival after TBI. In this process, the BCL2-interacting protein 3-like (BNIP3L/NIX)-mediated mitophagy mechanism ensures the clearance of damaged mitochondria and the limitation of oxidative stress [13]. However, BNIP3L-driven mitophagy can be adaptive or maladaptive depending on injury severity, timing, and cellular context. Accordingly, changes in BNIP3L expression may reflect both direct modulation of mitophagy and indirect effects secondary to altered injury burden.

Post-traumatic cellular hypoxia leads to increased expression of hypoxia-inducible factor-1 alpha (HIF-1α). While HIF-1α is involved in maintaining intracellular oxygen balance, its excessive activation can trigger microglial and astrocytic inflammation. This process contributes to the deepening of neuronal damage and the expansion of secondary brain injury. Therefore, modulation of HIF-1α is an important area of research for controlling the post-traumatic neuroinflammatory response [7].

Another molecular regulator involved in the progressive phase of TBI is the high mobility group box-1 (HMGB1) protein. This protein, released by neurons and glial cells, initiates immune signaling via toll-like receptor-4 (TLR) and receptor for advanced glycation end-products (RAGE) and can increase the severity of neuroinflammation [14,15]. Increased HMGB1 has been associated with BBB disruption, edema, and cell death. In this process, increases in proinflammatory cytokines, including tumor necrosis factor-alpha (TNF-α) and C-reactive protein (CRP), are noteworthy. TNF-α triggers apoptosis-related pathways by stimulating microglial activation, while CRP is considered an indicator of systemic inflammatory load [16]. In brain tissue, CRP immunoreactivity may reflect BBB leakage with the plasma protein extravasation and/or local expression by resident cells, and thus may serve as a surrogate marker of vascular-neuroinflammatory activation after TBI.

Furthermore, Caspase-3 (Cas-3) is one of the main enzymes responsible for neuronal apoptosis after trauma and has been found to be associated with the severity of injury in both experimental and clinical TBI studies [17]. Therefore, changes in inflammation and apoptosis markers such as TNF-α, CRP, and Cas-3 following ATD treatment are valuable biomarkers for understanding the drug’s neuroprotective effects.

All these data suggest that ATD is not only an NMDA antagonist but also a multitargeted neuroprotective compound that regulates oxidative stress, inflammation, *HIF-1α*, and *HMGB1*-mediated signaling pathways, as well as mitophagic mechanisms.

This study aims to evaluate the potential neuroprotective effects of ATD in a well-established rat TBI model. The study aims to investigate the regulatory effects of the drug on pathways related to oxidative stress (TOS, TAS, OSI), inflammation (CRP, TNF-α), apoptosis (Cas-3), and *HMGB1*; and to reveal the therapeutic potential of different application times in reducing secondary brain injury. The overall experimental workflow and molecular targets analyzed are summarized in the Graphical Abstract.

In this study, we show that post-traumatic ATD treatment improves oxidative balance (TOS/TAS/OSI), attenuates cortical histopathological damage and hemorrhage, and reduces inflammatory and apoptotic markers (TNF-α, CRP, Cas-3). In parallel, ATD was associated with lower *HIF-1α*, *BNIP3L*, and *HMGB1* gene expression in the cerebral cortex, supporting an association with suppressed secondary injury signatures across early and delayed treatment time points.

## 2. Materials and Methods

### 2.1. Ethical Approval

All experimental protocols were conducted in accordance with the ARRIVE v2.0 guidelines and were approved by the Süleyman Demirel University Animal Ethics Committee (Protocol No: 11.05.2023-166/05). Animal welfare principles were strictly adhered to in the experiments.

### 2.2. Animals and Experimental Design

The study used 32 adult male Wistar albino rats weighing between 250 and 300 g. The animals were housed in Euro Type 4 cages at 23 °C, 55% humidity, and a 12-h light/dark cycle. All animals had free access to standard pellet feed and water. The rats were aged 10–12 weeks at the time of TBI induction.

The rats were randomly divided into four groups of eight animals:

Group I (Sham): Animals that were anesthetized but not traumatized received 0.5–1 mL of physiological saline intraperitoneally (i.p.). They were sacrificed 24 h later.

Group II (Trauma): Trauma was induced using the impact-acceleration model described by Cikrik et al. In this model, a 50 g metal weight was dropped from a height of 80 cm onto the skull of anesthetized rats to induce diffuse brain injury [18]. After trauma, animals received 0.5–1 mL of physiological saline i.p. and were sacrificed 24 h later.

Group III (Trauma + ATD 1 day): A single dose of ATD (45 mg/kg, i.p.; A1260-5G, Sigma-Aldrich, St. Louis, MO, USA) was administered after trauma. Animals were sacrificed 24 h later.

Group IV (Trauma + ATD 7 days): The same dose of ATD (45 mg/kg/day, i.p.) was administered for 7 days after trauma, and the animals were sacrificed at the end of the seventh day.

The selected ATD dose of 45 mg/kg was determined based on previous studies [8,9] showing neuroprotective effects in TBI models. This dose provides a balanced therapeutic range between dopaminergic activation and NMDA antagonism.

Anesthesia during trauma induction and sacrifice was achieved using a combination of xylazine (10 mg/kg; Xylazin Bio 2%, Bioveta, Ivanovice na Hané, Czech Republic) and ketamine (90 mg/kg; Keta-Control, Doğa İlaç, Istanbul, Turkey). Sacrifice was performed under deep anesthesia by blood collection from the inferior vena cava. Brain tissue was removed without traumatization. A portion of the tissues was fixed in 10% buffered formalin for histological and immunohistochemical examination; the remaining portions were stored at −80 °C for biochemical and molecular analysis.

Brain region sampling: For biochemical assays (TOS/TAS/OSI) and qPCR, tissues were collected from the cerebral cortex. For histopathology and immunohistochemistry, paraffin blocks were prepared from coronal sections encompassing the cerebral cortex.

### 2.3. Trauma Model and Evaluation (Samples Were Taken from the Cerebral Cortex as Noted Above)

To ensure homogeneity of the trauma, each rat’s head was immobilized prior to impact, and no bone fracture or bleeding was observed at the application site. Post-trauma spontaneous resumption of breathing and reflex response times were recorded to standardize the model.

### 2.4. Biochemical Analysis

Brain tissues were homogenized by diluting them 1:9 (weight/volume) with phosphate-buffered saline (10 mM, pH 7.4) (IKA T25 Ultra-Turrax, IKA-Werke GmbH & Co., KG, Staufen, Germany). Homogenates were centrifuged at 2000 rpm for 20 min at 4 °C (NF 1200R, Nüve, Ankara, Turkey). The supernatants were used for total antioxidant level (TAS) and total oxidant level (TOS) analyses.

TAS and TOS levels were measured colorimetrically using an automatic biochemistry analyzer (AU5800, Beckman Coulter, Brea, CA, USA) according to the Erel method [11,17,18]. Results were expressed as µmol H_2_O_2_ equivalent/L for TOS and mmol Trolox equivalent/L for TAS.

The Oxidative Stress Index (OSI) was calculated using the formula (TOS/TAS) × 100 [19]. Total protein content was determined using the BCA method (562 nm), and results were normalized per mg of protein.

### 2.5. Histopathological Examination

Sections were obtained from the cerebral cortex for histopathological scoring.

Brain tissues were fixed in 10% buffered formalin, embedded in paraffin after routine tissue processing, and sectioned into 5 µm thick serial sections. Sections were stained with hematoxylin-eosin (H&E) and evaluated under a light microscope (CX41, Olympus, Tokyo, Japan).

Histopathological changes were graded from 0 to 4 according to the criteria defined by Mielke et al. [19] (Table 1).

The table evaluates the degree of deterioration in the meningeal and parenchymal structures according to the degree of subarachnoid hemorrhage.

### 2.6. Immunohistochemical (IHC) Analysis

Sections were mounted on poly-L-lysine-coated slides. IHC staining was performed using the streptavidin-biotin peroxidase method.

Primary antibodies:

TNF-α (Recombinant Anti-TNF-α [EPR21753-109], ab205587)

Cas-3 (Anti-Caspase-3 [EPR18297], ab184787)

CRP (Recombinant Anti-CRP [EPR23975-119], ab259862)

Were selected (Abcam, Cambridge, UK). All antibodies were diluted 1:100. The Mouse and Rabbit Specific HRP/DAB Detection Kit (ab236466, Abcam) was used as the secondary antibody. Image analysis was performed by two independent researchers in a group-blinded manner; consensus was reached in case of disagreements. In negative controls, the dilution buffer was applied instead of the primary antibody. Five non-overlapping areas were selected within the cerebral cortex for each rat; 20 cells were randomly evaluated at 40× magnification in each area (total 100 cells). The percentage of positively stained cells was calculated using ImageJ 1.46r (NIH, Bethesda, MD, USA) software.

Staining intensity was also evaluated using the semiquantitative H-score method. Images were captured using an Olympus CX41 microscope and CellSens Life Science Imaging software v2.1 (Olympus Corporation, Tokyo, Japan).

### 2.7. Genetic Analysis (qPCR) (RNA Was Extracted from the Cerebral Cortex Tissue Samples)

RNA was isolated from homogenized tissue samples using the GeneAll RiboEx™ Kit (GeneAll Biotechnology, Seoul, Republic of Korea) (Cat No: 301-001) according to the manufacturer’s instructions. RNA quantity and purity were measured using a NanoDrop UV-Vis spectrophotometer (Shimadzu UV-2600, Shimadzu Corporation, Kyoto, Japan).

cDNA synthesis was performed using 1 µg of RNA with the A.B.T.™ cDNA Synthesis Kit (Atlas Biotechnology, Ankara, Turkey) (Cat No: C03-01-05). Primer sequences were designed using the NCBI Primer-BLAST tool (https://www.ncbi.nlm.nih.gov/tools/primer-blast/, accessed on 9 February 2026).

The primer sequences and gene product lengths used are given in Table 2.

Real-time PCR was performed using the A.B.T.™ SYBR Master Mix (Cat No: Q04-01-05) on a Bio-Rad CFX96 (Bio-Rad Laboratories, Hercules, CA, USA) device. 18S ribosomal RNA (*Rn18s*) was used as the reference gene.

Each sample was run in triplicate. PCR conditions: 5 min pre-denaturation at 95 °C; followed by 40 cycles (15 s at 95 °C, 30 s at 55 °C).

Primer efficiencies ranged from 92% to 105%. Average Ct values ranged from 18 to 28. Relative gene expression was calculated using the 2^−ΔΔCt^ method. The melting curve showed a single peak; no amplification was observed in NTC samples.

### 2.8. Molecular Target Rationale

The selected molecular targets were chosen to interrogate specific mechanisms of secondary brain injury. *HIF-1α* represents hypoxia-driven transcriptional activation, *BNIP3L* (*NIX*) reflects mitophagy-related mitochondrial quality control, and *HMGB1* serves as a key damage-associated molecular pattern (DAMP) molecule linking mitochondrial stress to inflammation. TNF-α, CRP, and Cas-3 were assessed as downstream markers of inflammatory and apoptotic cascades. Together, these parameters delineate the hypoxia-mitophagy-inflammation axis relevant to TBI pathophysiology.

### 2.9. Statistical Analysis

Data were analyzed using GraphPad Prism v10.1 (GraphPad Software, San Diego, CA, USA) software. First, the Shapiro–Wilk test was used to assess distribution normality. Variables showing normal distribution (*p* > 0.05) were compared using one-way ANOVA. The Tukey post hoc test was used for pairwise analyses.

The significance level was set at *p* < 0.05. Results are expressed as mean ± standard deviation (SD).

A pre-a priori power analysis was performed using G*Power v3.1.9.4 software to determine the minimum sample size required for the study. Because TBI-specific pilot variance estimates were not available, we adopted an a priori large effect size assumption (f = 0.40) for a four-group one-way ANOVA, with α error probability 0.05 and power (1 − β) = 0.80. Under these assumptions, the required sample size was calculated as *n* = 28 (7 animals per group). To increase reliability and compensate for potential post-traumatic mortality, 32 rats (8 per group) were included in the study [20].

### 2.10. Study Design and Reporting

The study was randomized and controlled; animals were randomly allocated to groups. Investigators performing histopathological and immunohistochemical scoring were blinded to group allocation. Mortality and exclusion criteria were recorded.

## 3. Results

### 3.1. ATD Treatment Reduces Oxidative Stress After Trauma

In the trauma group, TOS and OSI values were significantly increased compared to the sham group (*p* = 0.012 and *p* = 0.002, respectively). Although the TAS decreased in the trauma group, this difference remained at the statistical threshold (*p* = 0.069).

One day of ATD administration resulted in a decrease in TOS and OSI values and an increase in TAS levels, but these changes did not reach the level of significance.

After seven days of treatment, oxidative balance improved significantly: TOS and OSI values decreased significantly (*p* = 0.041 and *p* < 0.001), while TAS levels increased significantly (*p* = 0.003).

**Figure 1:**
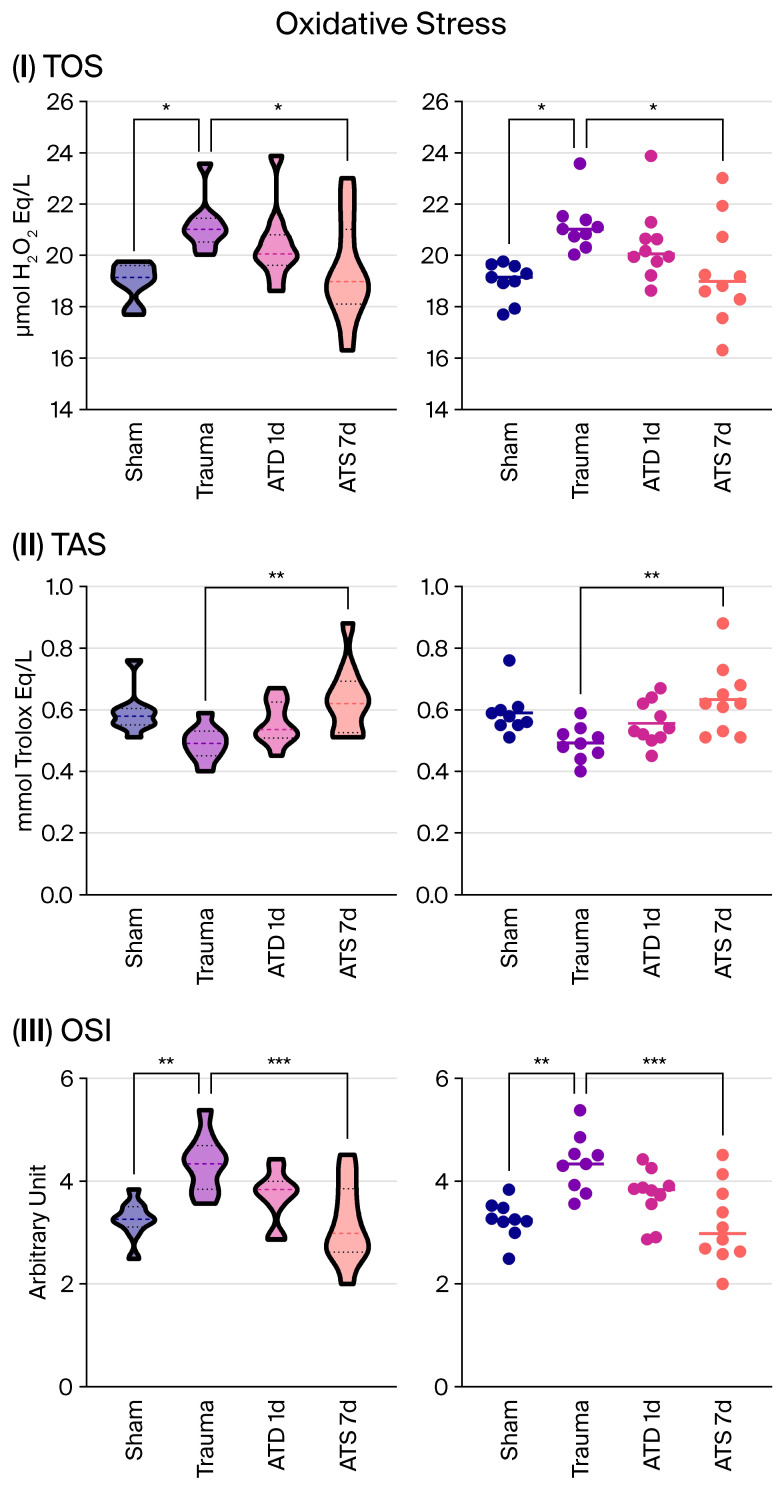
Oxidative stress parameters in the cerebral cortex following experimental traumatic brain injury (TBI). Total oxidant status (TOS), total antioxidant status (TAS), and oxidative stress index (OSI) are shown for sham, trauma, trauma + amantadine (ATD) (1 day), and trauma + ATD (7 days) groups. Individual values are shown as dots, with each dot representing the measurement obtained from a single animal. The horizontal line within each group indicates the group mean. “*” represents *p* < 0.05, “**” represents *p* < 0.01, and “***” represents *p* < 0.001.

These findings indicate that ATD significantly alleviates the oxidative stress response after trauma, particularly with long-term administration (Figure 1).

### 3.2. ATD Reduces Cortical Hemorrhage and Histopathological Damage

Histopathological examination revealed no significant pathology in the sham group except for mild hyperemia in the meningeal region. The trauma group showed widespread hemorrhage and tissue disorganization, and histopathological scores were significantly higher (*p* < 0.001).

Following ATD administration, hemorrhage was significantly reduced. After 1 day of treatment, hemorrhagic foci were significantly reduced (*p* < 0.001), and after 7 days of treatment, hemorrhagic areas were minimized, with signs of healing and localized fibrotic tissue formation observed (*p* < 0.001).

**Figure 2:**
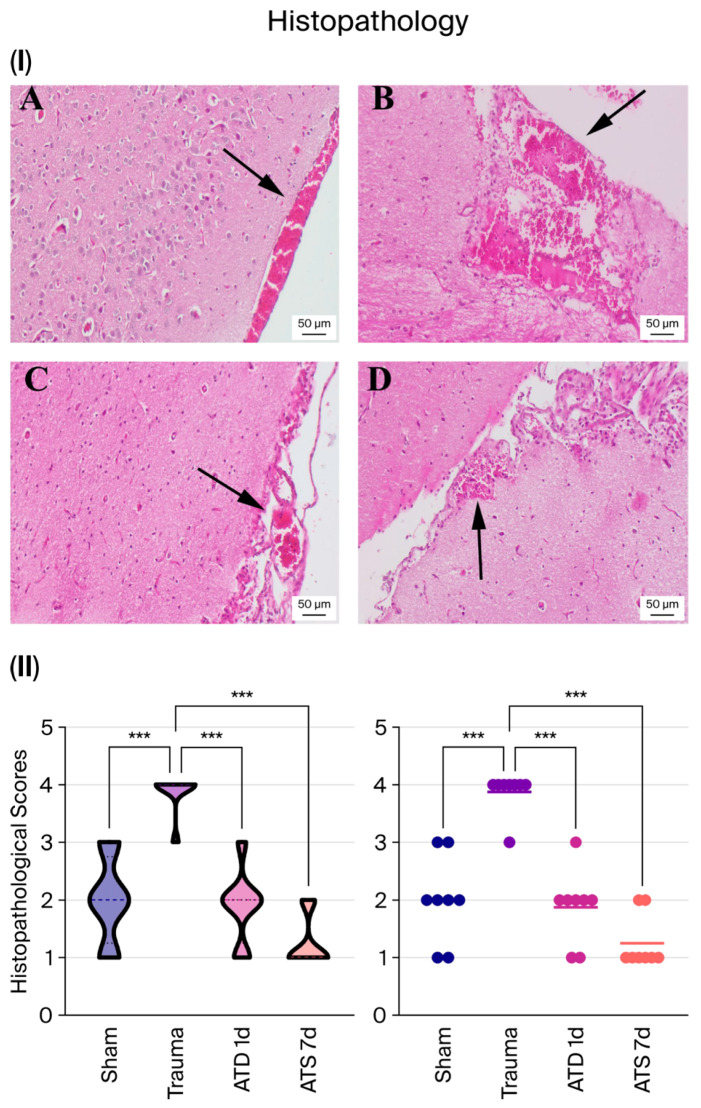
Histopathological evaluation of cortical injury following experimental TBI. Representative hematoxylin–eosin-stained sections (**I**) and semiquantitative histopathological scores (**II**) are shown for the sham, trauma, trauma + ATD (1 day), and trauma + ATD (7 days) groups. (**A**) Displays mild hyperemia alongside pronounced hemorrhage (indicated by arrowhead) in the meningeal area within the sham group. (**B**) Marked hyperemia with severe hemorrhage foci (arrow) in the brain of a rat from the trauma group. (**C**) A significantly reduced hemorrhage area (arrow) was detected in the 1-day treatment group. (**D**) A notable decrease in the hemorrhagic area was accompanied by increased fibrotic tissue in a rat from the ATD 7d group. The scale bars represent 50 μm. The relationships between groups (n = 8 for each) were assessed by one-way ANOVA and post hoc Tukey’s test. Individual values are shown as dots, with each dot representing the measurement obtained from a single animal. The horizontal line within each group indicates the group mean. “***” represents *p* < 0.001.

According to histopathological scoring, ATD administration reduced tissue damage (Figure 2).

### 3.3. Immunohistochemical Analyses Show That Inflammation and Apoptosis Are Suppressed

Immunohistochemical studies showed that CRP, Cas-3, and TNF-α expressions were significantly increased in the trauma group compared to the sham group (*p* < 0.001 for all three).

CRP: Decreased significantly with both 1-day and 7-day ATD treatment (*p* < 0.001 for both). After 7 days of treatment, CRP expression decreased to sham levels.

Cas-3: Decreased significantly in both treatment groups (*p* < 0.001 for both). While one-day treatment resulted in normalization close to sham levels, expression was notably lower than in the sham group after seven days of treatment (*p* = 0.015).

TNF-α: Expression levels decreased significantly with ATD treatment in both treatment groups (*p* < 0.001 for both). After 7 days of treatment, TNF-α levels were not statistically different from those in the sham group (*p* = 0.583).

**Figure 3:**
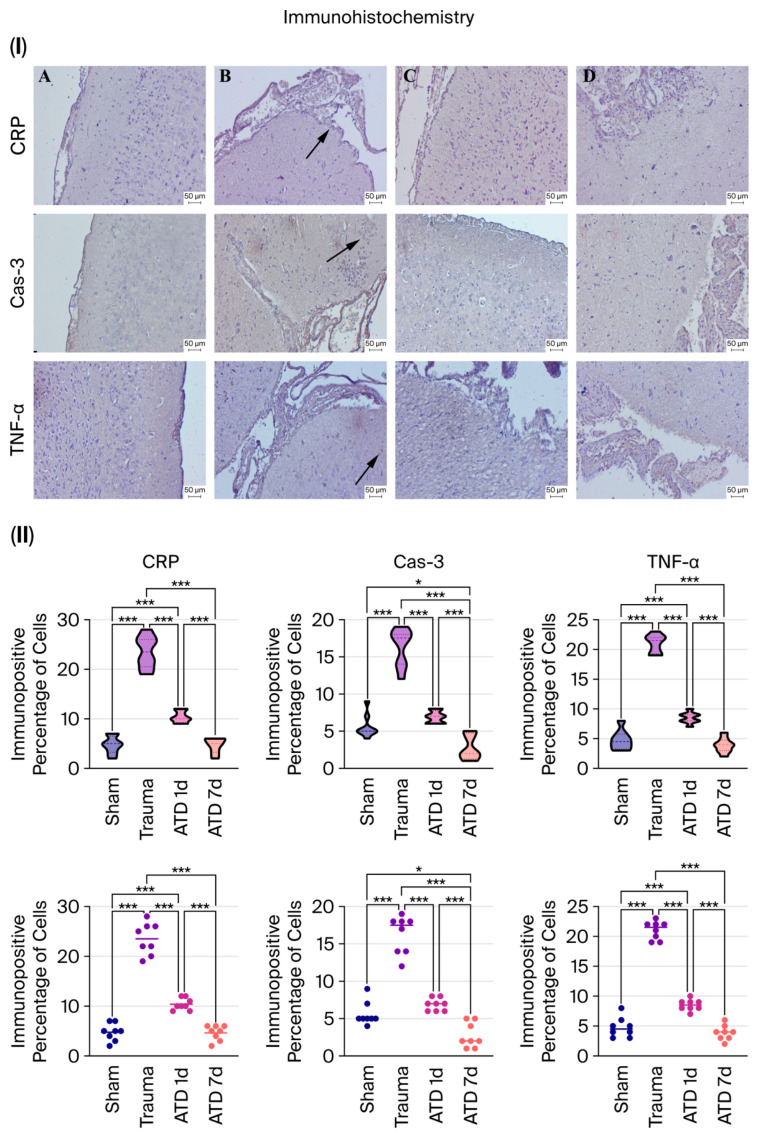
Immunohistochemical assessment of inflammatory and apoptotic markers in the cerebral cortex. Representative staining (**I**) and quantitative analysis (**II**) of C-reactive protein (CRP), tumor necrosis factor alpha (TNF-α), and caspase-3 (Cas-3) are shown across study groups. Negative controls were processed without the primary antibody. (**A**) Negative results for slight expression of CRP, Cas-3, and TNF-α in the sham group. (**B**) Indicates a significant increase in CRP, Cas-3, and TNF-α expression (marked by arrows) in the trauma group. (**C**) A marked decrease in CRP, Cas-3, and TNF-α expression was detected in the 1-day group. (**D**) CRP, Cas-3, and TNF-α expression were not detected in the 7-day group. These images were obtained via the streptavidin–biotin peroxidase method, with scale bars representing 50 μm. The relationships between groups (n = 8 for each) were assessed by one-way ANOVA and post hoc Tukey’s test. Individual values are shown as dots, with each dot representing the measurement obtained from a single animal. The horizontal line within each group indicates the group mean. “*” represents *p* < 0.05 and “***” represents *p* < 0.001.

These data indicate that ATD effectively suppresses the inflammatory response and apoptotic processes in a time-dependent manner (Figure 3).

### 3.4. ATD Suppresses Gene Expression Associated with Secondary Damage

In qPCR analyses at the molecular level, *BNIP3L*, *HIF-1α*, and *HMGB1* gene expression was significantly increased in the trauma group compared to the sham group (*p* < 0.001 for all three).

*BNIP3L*: This increase was significantly reduced with ATD treatment in both 1-day and 7-day applications (*p* < 0.001 for both). After 7 days of treatment, *BNIP3L* expression approached sham levels but remained slightly elevated (*p* = 0.018).

*HIF-1α*: A significant decrease was observed in both treatment groups (*p* < 0.001 for both). There was no difference between short- and long-term treatments (*p* = 0.527).

*HMGB1*: Compared to the trauma group, expressions were significantly decreased in both treatment groups (*p* < 0.001 for both). 

**Figure 4:**
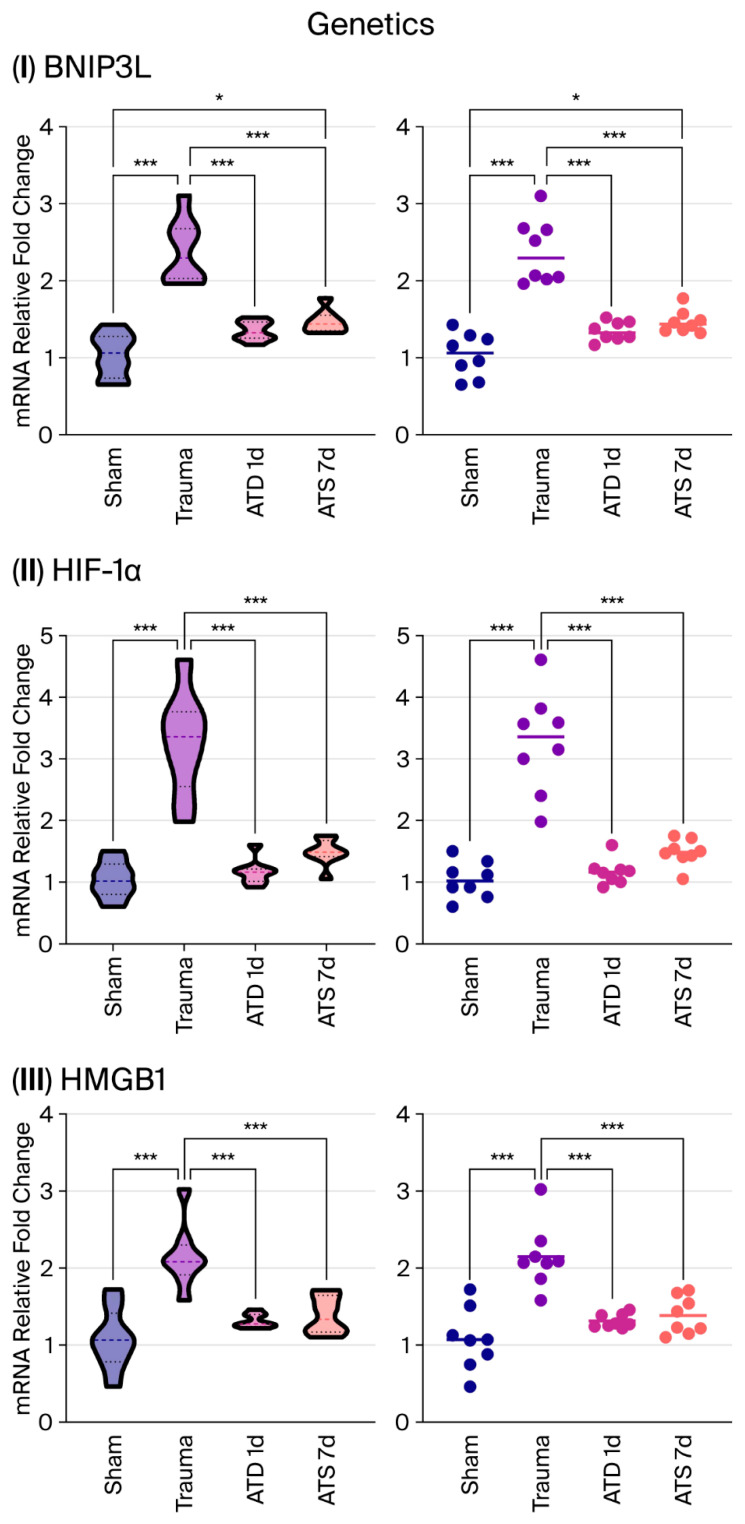
qPCR-based relative gene expression in the cerebral cortex following experimental TBI. Relative mRNA expression levels of *HIF-1α*, *BNIP3L*, and *HMGB1* are shown across sham, trauma, trauma + ATD (1 day), and trauma + ATD (7 days) groups. “*” represents *p* < 0.05 and “***” represents *p* < 0.001.

These findings indicate that ATD treatment is associated with lower mRNA expression of *BNIP3L*, *HIF-1α*, and *HMGB1* after TBI, consistent with attenuation of secondary injury-related transcriptional responses; however, mitophagy was not directly assessed, and these gene-expression changes were not validated at the protein level (Figure 4).

These findings indicate that ATD suppresses the molecular mechanisms of secondary brain injury by regulating the expression of genes associated with mitophagy (*BNIP3L*), the hypoxia response (*HIF-1α*), and inflammation-mediated (*HMGB1*) pathways (Figure 4).

Overall, ATD reduced oxidative stress, inflammation, apoptosis, and injury-associated changes in gene expression in a time-dependent manner after TBI. Short-term treatment provided partial improvement in biochemical parameters, while seven days of administration produced more pronounced improvements in biochemical and histological readouts.

## 4. Discussion

TBI remains a serious global health problem due to high mortality and morbidity rates, as well as long-term neurocognitive impairments, behavioral changes, and physical sequelae [21,22]. Secondary brain injury is a complex process involving the interaction of multiple mechanisms, including cytotoxicity, oxidative stress, inflammation, hypoxia, and mitochondrial dysfunction [23,24]. Therefore, therapeutic strategies targeting multiple steps of the pathophysiology are of great importance for neuroprotection.

In this study, the protective potential of ATD ATD, known for its NMDA receptor antagonism and dopaminergic activity modulation, against secondary brain injury after trauma was investigated. Overall, our findings suggest that ATD attenuates oxidative stress and inflammatory/apoptotic responses and is associated with concurrent changes in the expression of *HMGB1*, *HIF-1α*, and *BNIP3L*. Importantly, these molecular changes should be interpreted as mechanistic correlations within the proposed hypoxia–mitophagy–inflammation framework rather than as definitive evidence of direct pathway modulation.

### 4.1. Oxidative Stress and Antioxidant Response

Increased ROS production and inadequate antioxidant defense after TBI are among the most important determinants of neuronal loss. In our study, we found a significant increase in TOS and OSI levels and a decrease in TAS levels in the trauma group. However, 7 days of ATD treatment significantly corrected this imbalance. Gündüz et al. [25] demonstrated that ATD reduced TOS levels and increased TAS in a cerebral ischemia–reperfusion model. Similarly, it has been reported that a decrease in total antioxidant capacity in critically ill TBI patients is associated with secondary organ damage [23]. Restoring antioxidant capacity is a fundamental goal in terms of preserving mitochondrial stability and preventing neuronal death [24]. These results suggest that prolonged ATD administration may alleviate secondary damage by reestablishing redox homeostasis.

### 4.2. Histopathological Improvement and Suppression of the Inflammatory Response

Histopathological findings supported the biochemical data and showed that ATD significantly reduced vascular congestion and hemorrhagic areas. Immunohistochemical analyses revealed that ATD significantly reduced post-traumatic increases in CRP, Cas-3, and TNF-α expression. Because CRP is primarily a systemic inflammatory marker, brain CRP immunoreactivity in this setting likely reflects BBB disruption and extravasation of circulating CRP and/or local tissue-associated CRP signal within inflamed vasculature and parenchyma; therefore, we interpreted CRP staining as a composite indicator of post-traumatic vascular-neuroinflammatory activity rather than purely systemic inflammation. This result is consistent with previous studies supporting the anti-inflammatory and anti-apoptotic effects of ATD [7,26]. Unluer et al. [26] reported that long-term ATD administration in a rabbit spinal ischemia model reduced Cas-3 activity and inflammatory markers and increased neuronal survival. Similarly, Wang et al. [27] demonstrated that 16 days of ATD treatment in a TBI model suppressed microglial activation, reduced TNF-α levels, and supported cognitive recovery. Our findings indicate that short-term treatment initiates an anti-inflammatory response, while longer administration is associated with more persistent attenuation of neuroinflammatory cascades.

### 4.3. Regulation of HMGB1-Mediated Neuroinflammation

In our study, *HMGB1* gene expression was significantly increased after trauma, and ATD treatment markedly reduced this increase. *HMGB1* is a potent proinflammatory DAMP protein released by necrotic neurons and activated glia. Once released, it binds to TLR4 and RAGE receptors, triggering microglial activation and cytokine release [14,28,29]. Redox imbalance deepens the inflammatory response by stimulating the translocation of *HMGB1* from the nucleus to the cytoplasm and its secretion [30,31]. This link between oxidative stress and *HMGB1* expression parallels the increase in TOS/OSI observed in our study.

The decrease in TOS and OSI following ATD treatment should be evaluated in conjunction with the normalization of *HMGB1* expression; this suggests that the antioxidant effects of ATD indirectly suppress *HMGB1*-mediated inflammation. Similarly, it has been reported that glycyrrhizin application reduces oxidative stress by inhibiting *HMGB1* activity and accelerates neurological recovery [32]. *HMGB1* is a primary regulator of neuroinflammation and brain edema, and *HMGB1*-neutralizing antibodies or A-box peptide analogues have been shown to reduce edema and tissue loss in TBI models [33,34]. Furthermore, pregabalin administration has been shown to suppress *HMGB1* translocation and glial activation in a radiation-induced brain injury model [35]. These data support the notion that *HMGB1* plays a central role in various neurodegenerative processes and that ATD may exert a regulatory effect on this pathway.

### 4.4. Hypoxia Response and HIF-1α Relationship

Hypoxic signaling pathways, particularly through *HIF-1α*, play an important role in secondary brain injury. *HIF-1α* is activated in the presence of oxygen deficiency and contributes to tissue remodeling and the persistence of the inflammatory response by increasing the expression of genes such as vascular endothelial growth factor [7,36,37,38]. In our study, the significantly increased *HIF-1α* expression in the trauma group was suppressed by ATD treatment. This finding is consistent with studies reporting that *HIF-1α* inhibition reduces edema and prevents neurological impairment after TBI [38]. *HIF-1α* also links hypoxia to inflammation by stimulating the production of proinflammatory cytokines such as TNF-α and IL-1β [7]. Therefore, ATD may also have weakened these upstream inflammatory pathways by reducing *HIF-1α* expression.

There is a bidirectional interaction between hypoxia, oxidative stress, and *HMGB1*. ROS production triggers the transition of *HMGB1* from the nucleus to the cytoplasm and its secretion [39], while extracellular *HMGB1* can also increase *HIF-1α* activation via the RAGE/NF-κB pathway [40]. This reciprocal cycle creates a positive feedback mechanism that enhances the secondary inflammatory response. In our study, the simultaneous decrease in both *HIF-1α* and *HMGB1* levels after ATD treatment suggests that this harmful interaction is broken.

### 4.5. Mitochondrial Dysfunction and BNIP3L-Mediated Mitophagy

Impaired mitochondrial integrity is one of the most important determinants of secondary neuronal damage. Following TBI, mitochondria fail to sustain energy production, leading to ROS accumulation and apoptosis [41]. Mitochondrial quality is maintained by selective autophagic mechanisms known as mitophagy [42]. The BNIP3L/NIX protein plays a critical role in this process; increased BNIP3L expression under hypoxic or oxidative stress conditions facilitates the elimination of damaged mitochondria [13,43]. However, excessive BNIP3L activation can lead to mitochondrial depletion and cellular energy deficiency [44]. Importantly, mitophagy was not directly measured in this study (e.g., LC3-II, p62, Parkin, mitochondrial turnover markers), and *BNIP3L* mRNA levels should not be interpreted as a measure of mitophagic flux.

In rodent TBI models, increased BNIP3L expression in the cortex and hippocampus has been associated with increased autophagic activity and neuronal loss [45]. Furthermore, altered BNIP3L/NIX signaling has been linked to mitochondrial dysfunction in ischemic stroke and epilepsy models [46]. In our study, *BNIP3L* expression was significantly increased in the trauma group, while this increase was markedly reduced with ATD treatment. Given that *BNIP3L*-mediated mitophagy may be protective (by removing damaged mitochondria) or deleterious (if excessive or coupled to cell death pathways) depending on context, the observed reduction in *BNIP3L* should be interpreted cautiously. It may reflect decreased activation of injury-driven mitochondrial stress responses, secondary to reduced overall tissue damage rather than direct pharmacological suppression of mitophagy per se. Future studies assessing mitophagy flux and employing genetic/pharmacologic manipulation of *BNIP3L* would be needed to establish causality. Nonetheless, the parallel improvements in oxidative stress indices and apoptosis markers are consistent with improved mitochondrial homeostatic balance in ATD-treated animals.

### 4.6. Proposed Mechanistic Framework

**Figure 5:**
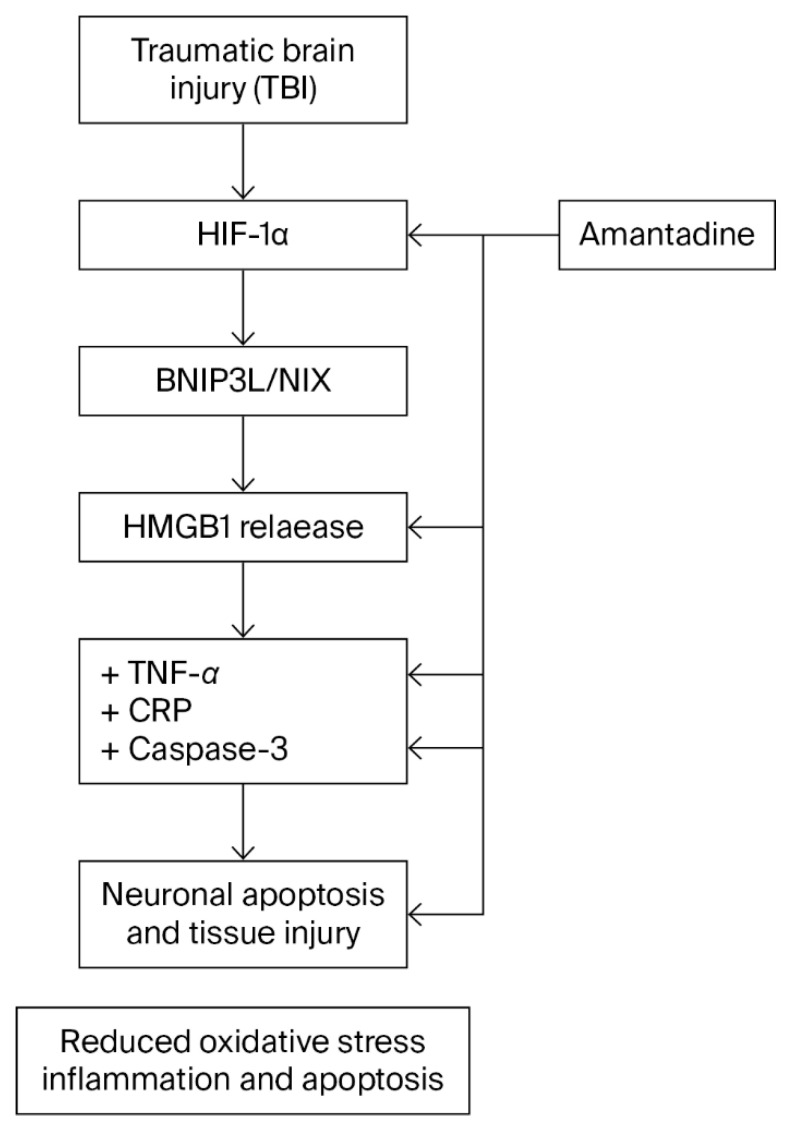
Proposed mechanistic framework of ATD-associated modulation of secondary injury after TBI. Schematic illustration summarizing the putative hypoxia–mitochondrial stress/mitophagy–DAMP-mediated inflammation axis in the cerebral cortex following TBI. Post-traumatic activation of hypoxia signaling (*HIF-1α*), mitochondrial stress–related responses (*BNIP3L*), and DAMP signaling (*HMGB1*) is shown in parallel with downstream inflammatory and apoptotic markers. ATD treatment is depicted as being associated with coordinated reductions in these molecular signatures, together with attenuation of oxidative stress and inflammatory/apoptotic responses.

Experimental TBI initiates a molecular response in which hypoxia signaling (*HIF-1α*), mitochondrial stress/mitophagy-related markers (*BNIP3L*), and DAMP-related inflammation (*HMGB1*) may rise in parallel with downstream inflammatory and apoptotic readouts such as TNF-α and Cas-3. In our dataset, ATD treatment was associated with coordinated reductions across these markers, consistent with dampening of a feed-forward hypoxia–mitochondrial stress–DAMP cycle. However, because this study did not include pathway-specific perturbations, the relationships within the proposed axis should be considered associative. By preserving histological integrity and reducing inflammatory signaling, ATD appears to act beyond NMDA antagonism in this model, but future work should directly test pathway dependencies to confirm mechanistic modulation. The proposed cascade and the pattern of changes observed with ATD are illustrated in Figure 5.

### 4.7. Limitations of the Study

Although the impact-acceleration model used in this study represents a well-defined experimental form of TBI, it may not fully reflect the multifocal and heterogeneous injury pattern seen in humans. Importantly, the absence of neurological/behavioral assessments substantially limits translational interpretation; therefore, our findings should be viewed as molecular and histological evidence consistent with neuroprotection rather than proof of functional recovery. Furthermore, using only male rats precludes any comments on sex differences. Future studies should evaluate the long-term effects of ATD by correlating molecular changes (including *HIF-1α*/*BNIP3L*/*HMGB1*-related markers) with validated behavioral and neurological outcome measures. Additionally, the proposed *HIF-1α*/*BNIP3L*/*HMGB1* framework is supported only by qPCR (mRNA) data, and protein-level confirmation (e.g., Western blot/ELISA) was not performed. Moreover, mitophagy was not directly assessed, and additional autophagy/mitophagy markers were not measured.

## 5. Conclusions

In summary, ATD improved oxidative balance and reduced histopathological injury, inflammatory staining, and apoptotic markers after experimental TBI. ATD treatment was also associated with lower *HIF-1α*, *BNIP3L*, and *HMGB1* expression, consistent with reduced secondary injury signatures within a hypoxia–mitochondrial stress/mitophagy–DAMP inflammation framework. However, these axis-related inferences are based on mRNA expression and should be interpreted cautiously in the absence of protein-level confirmation and direct mitophagy measurements. Because neurological/behavioral outcomes and pathway-specific causal tests were not performed, these findings should be interpreted as evidence of molecular and histological attenuation consistent with neuroprotection rather than definitive proof of direct axis modulation or functional recovery. Further studies incorporating functional endpoints and mechanistic interventions are warranted. Specifically, compared with the untreated trauma group, ATD reduced oxidant burden and improved antioxidant status, decreased cortical hemorrhage/tissue disruption, and lowered TNF-α, CRP, and Cas-3 immunoreactivity, while being associated with reduced *HIF-1α*, *BNIP3L*, and *HMGB1* mRNA expression in the cerebral cortex.

## Figures and Tables

**Table 1:** Histopathological scores of subarachnoid hemorrhages.

0	Normal meningeal and parenchymal structure
1	No blood in the subarachnoid space, ventricles, or brain parenchyma.
2	No localized or diffuse thin subarachnoid hemorrhage, intraventricular, or intraparenchymal hemorrhage.
3	No diffuse or localized thick subarachnoid blood layers, intraventricular, or intraparenchymal hemorrhage.
4	Intraventricular or intraparenchymal hemorrhage in association with subarachnoid hemorrhage, regardless of thickness or location.

**Table 2:** Primary sequences, product sizes, and accession numbers of genes.

Genes	Primary Sequence	Product Size	Accession Number
*Rn18s* (Housekeeping)	F: CTCTAGATAACCTCGGGCCG	209 bp	NR_046237.2
*Rn18s* (Housekeeping)	R: GTCGGGAGTGGGTAATTTGC	209 bp	NR_046237.2
*BNIP3L*	F: TTTAAAGCAGCTCTGGAGCCC	185 bp	NM_080888.2
*BNIP3L*	R: GGCCTGAGACACTCCTTACA	185 bp	NM_080888.2
*HMGB1*	F: GCGCTTTTGTGATGGAGTGC	244 bp	NM_012963.3
*HMGB1*	R: GCACCAAGTGTTGTTAATGGGG	244 bp	NM_012963.3
*HIF 1 α*	F: GCAACTAGGAACCCGAACCA	251 bp	NM_024359.2
*HIF 1 α*	R: TCGACGTTCGGAACTCATCC	251 bp	NM_024359.2

F: Forward, R: Reverse, *Rn18s*: 18S ribosomal RNA, *BNIP3L*: BCL2 Interacting Protein 3-Like, *HMGB1*: High mobility group box 1, *HIF1 α*: Hypoxia-inducible factor-1 alpha.

## Data Availability

The datasets generated and/or analyzed during the current study are available from the corresponding author upon reasonable request.

## Abbreviations

The following abbreviations are used in this manuscript:

ATD	Amantadine
BBB	Blood–brain barrier
BNIP3L	BCL2/adenovirus E1B 19 kDa protein-interacting protein 3-like
Cas-3	Caspase-3
cDNA	Complementary deoxyribonucleic acid
CRP	C-reactive protein
DAMP	Damage-associated molecular pattern
H&E	Hematoxylin and eosin
HIF-1α	Hypoxia-inducible factor-1 alpha
HMGB1	High mobility group box-1
IHC	Immunohistochemistry
IL	Interleukin
NF-κB	Nuclear factor kappa B
NIX	Nip-like protein X (alternate name for BNIP3L)
NMDA	N-methyl-D-aspartate
OSI	Oxidative Stress Index
PCR	Polymerase chain reaction
qPCR	Quantitative polymerase chain reaction
RAGE	Receptor for advanced glycation end-products
RNA	Ribonucleic acid
ROS	Reactive oxygen species
TAS	Total Antioxidant Status
TBI	Traumatic brain injury
TLR	Toll-like receptor
TNF-α	Tumor necrosis factor-alpha
TOS	Total Oxidant Status

## References

[1] James S.L., Theadom A., Ellenbogen R.G., Bannick M.S., Montjoy-Venning W., Lucchesi L.R., Abbasi N., Abdulkader R., Abraha-Niguse H., Adsuar J.C. (2019). Global, regional, and national burden of traumatic brain injury and spinal cord injury, 1990–2016: A systematic analysis for the Global Burden of Disease Study 2016. Lancet Neurol..

[2] Haagsma J.A., Graetz N., Bolliger I., Naghavi M., Higashi H., Mullany E.C., Ameh A.E., Ammar W., Barrero H.L., Bekele T. (2016). The global burden of injury: Incidence, mortality, disability-adjusted life years and time trends from the Global Burden of Disease Study 2013. Inj. Prev..

[3] Taylor C.A., Bell J.M., Breiding M.J., Xu L. (2017). Traumatic brain injury-related emergency department visits, hospitalizations, and deaths—United States, 2007 and 2013. MMWR Surveill. Summ..

[4] Prins M.L., Alexander D., Giza C.C., Hovda D.A. (2013). Repeated mild traumatic brain injury: Mechanisms of cerebral vulnerability. J. Neurotrauma.

[5] Simon D.W., McGeachy M.J., Bayır H., Clark R.S., Loane D.J., Kochanek P.M. (2017). The far-reaching scope of neuroinflammation after traumatic brain injury. Nat. Rev. Neurol..

[6] Ribeiro F.C.P., de Oliveira N.V., Coral G.R., de Assis César A.R., Gonçalves M.W.A., Egal E.S.A., Pereira F.K. (2025). Efficacy of N-methyl-D-aspartate receptor antagonists in treating traumatic brain injury-induced brain edema: A systematic review and meta-analysis of animal studies. Neurocrit. Care.

[7] Xu X., Yang M., Zhang B., Dong J., Zhuang Y., Ge Q., Niu F., Liu B. (2023). HIF-1α participates in secondary brain injury through regulating neuroinflammation. Transl. Neurosci..

[8] Danysz W., Dekundy A., Scheschonka A., Riederer P. (2021). Amantadine: Reappraisal of the timeless diamond—Target updates and novel therapeutic potentials. J. Neural Transm..

[9] Dekundy A., Pichler G., El Badry R., Scheschonka A., Danysz W. (2024). Amantadine for traumatic brain injury—Supporting evidence and mode of action. Biomedicines.

[10] Di Pietro V., Yakoub K.M., Caruso G., Lazzarino G., Signoretti S., Barbey A.K., Tavazzi B., Lazzorino G., Belli A., Amorini M.A. (2020). Antioxidant therapies in traumatic brain injury. Antioxidants.

[11] Erel O. (2004). A novel automated direct measurement method for total antioxidant capacity using a new generation, more stable ABTS radical cation. Clin. Biochem..

[12] Valko M., Leibfritz D., Moncol J., Cronin M.T.D., Mazur M., Telser J. (2007). Free radicals and antioxidants in normal physiological functions and human disease. Int. J. Biochem. Cell. Biol..

[13] Li Y., Zheng W., Lu Y., Zheng Y., Pan L., Wu X., Yuan Y., Shen Z., Ma S., Zhang X. (2022). BNIP3L/NIX-mediated mitophagy: Molecular mechanisms and implications for human disease. Cell Death Dis..

[14] Paudel Y.N., Shaikh M.F., Chakraborti A., Kumari Y., Aledo-Serrano Á., Aleksovska K., Alvim M.K.M., Othman I. (2018). HMGB1: A common biomarker and potential target for TBI, neuroinflammation, epilepsy, and cognitive dysfunction. Front. Neurosci..

[15] Wang P., Okada-Rising S., Scultetus A.H., Bailey Z.S. (2024). The relevance and implications of monoclonal antibody therapies on traumatic brain injury pathologies. Biomedicines.

[16] Arand M., Melzner H., Kinzl L., Bruckner U.B., Gebhard F. (2001). Early inflammatory mediator response following isolated traumatic brain injury and other major trauma in humans. Langenbecks Arch. Surg..

[17] Kajta M. (2004). Apoptosis in the central nervous system: Mechanisms and protective strategies. Pharmacol. Rep..

[18] Cikriklar H.I., Onur U., Ekici M.A., Ozbek Z., Cosan T.D., Yucel M., Yurumwz Y., Baydemir C. (2015). Effectiveness of GFAP in determining neuron damage in rats with induced head trauma. Turk. Neurosurg..

[19] Mielke D., Bleuel K., Stadelmann C., Rothe V., Malinova V. (2020). The ESAS-score: A histological severity grading system of subarachnoid hemorrhage using the modified double hemorrhage model in rats. PLoS ONE.

[20] Faul F., Erdfelder E., Lang A.-G., Buchner A. (2007). G*Power 3: A flexible statistical power analysis program for the social, behavioral, and biomedical sciences. Behav. Res. Methods.

[21] Lipsky R.H., Witkin J.M., Shafique H., Smith L.J., Cerne R., Marini M.A. (2024). Traumatic brain injury: Molecular biomarkers, genetics, secondary consequences, and medical management. Front. Neurosci..

[22] Cieri M.B., Ramos A.J. (2025). Astrocytes, reactive astrogliosis, and glial scar formation in traumatic brain injury. Neural Regen. Res..

[23] Rynkiewicz-Szczepanska E., Kosciuczuk U., Maciejczyk M. (2024). Total antioxidant status in critically ill patients with traumatic brain injury and secondary organ failure: A systematic review. Diagnostics.

[24] Zhao P., Shi W., Ye Y., Xue K., Hu J., Chao H., Tao Z., Xu L., Gu W., Zhang L. (2024). Atox1 protects hippocampal neurons after traumatic brain injury via DJ-1-mediated anti-oxidative stress and mitophagy. Redox Biol..

[25] Gündüz Z.B., Aktaş F., Vatansev H., Solmaz M., Erdogan E. (2021). Effects of amantadine and topiramate on neuronal damage in rats with experimental cerebral ischemia-reperfusion. Adv. Clin. Exp. Med..

[26] Unluer C., Kuru Bektasoglu P., Erguder B.İ., Arikok T.A., Ermutlu I., Gurer B., Kertmen H. (2024). Amantadine’s neuroprotective effects in a rabbit spinal cord ischemia/reperfusion model. Turk. Neurosurg..

[27] Wang T., Huang X.J., Van K.C., Went T.G., Nguyen T.J., Lyeth G.B. (2014). Amantadine improves cognitive outcome and increases neuronal survival after fluid percussion traumatic brain injury in rats. J. Neurotrauma.

[28] Prantner D., Nallar S., Vogel S.N. (2020). The role of RAGE in host pathology and crosstalk between RAGE and TLR4 in innate immune signal transduction pathways. FASEB J..

[29] Paudel Y.N., Angelopoulou E., Piperi C., Othman I., Shakih F.M. (2020). HMGB1-mediated neuroinflammatory responses in brain injuries: Potential mechanisms and therapeutic opportunities. Int. J. Mol. Sci..

[30] Tang D., Kang R., Livesey K.M., Zeh J.H., Lotze T.M. (2011). High mobility group box 1 (HMGB1) activates an autophagic response to oxidative stress. Antioxid. Redox Signal..

[31] Janko C., Filipović M., Munoz L.E., Schorn C., Schett G., Burmazovic I.I., Herrmann M. (2014). Redox modulation of HMGB1-related signaling. Antioxid. Redox Signal..

[32] Zhu K., Zhu X., Liu S., Jie Y., Wu S., Hei M. (2022). Glycyrrhizin attenuates hypoxic-ischemic brain damage by inhibiting ferroptosis and neuroinflammation in neonatal rats via the HMGB1/GPX4 pathway. Oxid. Med. Cell. Longev..

[33] Yang L., Wang F., Yang L., Yuan Y., Chen Y., Zhang G., Fan Z. (2018). HMGB1 A-box reverses brain edema and deterioration of neurological function in a traumatic brain injury mouse model. Cell Physiol. Biochem..

[34] Denorme F., Portier I., Rustad J.L., Cody J.M., De Araujo V.C., Hoki C., Alexander D.M., Grandhi R., Dyer R.M., Neal M.D. (2022). Neutrophil extracellular traps regulate ischemic stroke brain injury. J. Clin. Investig..

[35] Zhang Z., Jiang J., He Y., Cai J., Xie J., Wu M., Xing M., Zhang Z., Chang H., Yu P. (2022). Pregabalin mitigates microglial activation and neuronal injury by inhibiting HMGB1 signalling pathway in radiation-induced brain injury. J. Neuroinflammation.

[36] Thelin E.P., Frostell A., Mulder J., Mulder J., Mitsios N., Damberg P., Aski N.S., Risling M., Svenson M., Kossman-Morganti C.M. (2016). Lesion size is exacerbated in hypoxic rats whereas HIF-1α and VEGF increase in injured normoxic rats: A prospective cohort study of secondary hypoxia in focal traumatic brain injury. Front. Neurol..

[37] Shenaq M., Kassem H., Peng C., Schafer S., Ding Y.J., Fredrickson V., Guthikonda M., Kreipke W.C., Rafols A.J., Ding Y. (2012). Neuronal damage and functional deficits are ameliorated by inhibition of aquaporin and HIF-1α after traumatic brain injury. J. Neurol. Sci..

[38] Bae Y.H., Joo H., Bae J., Hyeon J.S., Her S., Ko E., Choi G.H., Hur M.E., Bu Y., Lee B.D. (2018). Brain injury induces HIF-1α-dependent transcriptional activation of LRRK2 that exacerbates brain damage. Cell Death Dis..

[39] Qiu B., Yuan P., Du X., Jin H., Du J., Huang Y. (2023). Hypoxia-inducible factor-1α is an important regulator of macrophage biology. Heliyon.

[40] Liu J., Wei E., Wei J., Zhou W., Webster A.K., Zhang B., Li D., Zhang G., Wei Y., Long Y. (2021). MiR-126-HMGB1-HIF-1 axis regulates endothelial cell inflammation during exposure to hypoxia-acidosis. Dis. Markers.

[41] Zhang S., Wu X., Wang J., Shi Y., Hu Q., Chui W., Bai H., Zhou J., Du Y., Han L. (2022). Adiponectin/AdipoR1 signaling prevents mitochondrial dysfunction and oxidative injury after traumatic brain injury in a SIRT3-dependent manner. Redox Biol..

[42] Lizama B.N., Chu C.T. (2021). Neuronal autophagy and mitophagy in Parkinson’s disease. Mol. Aspects Med..

[43] Wu X., Zheng Y., Liu M., Li Y., Ma S., Tang W., Yan W., Cao M., Zheng W., Jiang L. (2021). BNIP3L/NIX degradation leads to mitophagy deficiency in ischemic brains. Autophagy.

[44] Gao K., Chen Y., Mo R., Wang C. (2024). Excessive BNIP3- and BNIP3L-dependent mitophagy underlies the pathogenesis of FBXL4-mutated mitochondrial DNA depletion syndrome. Autophagy.

[45] Ma J., Ni H., Rui Q., Liu H., Jiang F., Gao R., Gao Y., Li D., Chen G. (2019). Potential roles of NIX/BNIP3L pathway in rat traumatic brain injury. Cell Transplant..

[46] Huang Q., Yu X., Fu P., Wu M., Yin X., Chen Z., Zhang M. (2024). Mechanisms and therapeutic targets of mitophagy after intracerebral hemorrhage. Heliyon.

